# Symptom Burden and Time from Symptom Onset to Cancer Diagnosis in Patients with Early-Onset Colorectal Cancer: A Multicenter Retrospective Analysis

**DOI:** 10.3390/curroncol31040158

**Published:** 2024-04-08

**Authors:** Victoria A. Baronas, Arif A. Arif, Eric Bhang, Gale K. Ladua, Carl J. Brown, Fergal Donnellan, Sharlene Gill, Heather C. Stuart, Jonathan M. Loree

**Affiliations:** 1 Division of Gastroenterology, University of British Columbia, Vancouver, BC V6T1Z4, Canada; v.baronas@alumni.ubc.ca (V.A.B.); arifarif@student.ubc.ca (A.A.A.); eric.bhang@bccancer.bc.ca (E.B.); gale.ladua@bccancer.bc.ca (G.K.L.); cbrown@providencehealth.bc.ca (C.J.B.); fergal.donnellan@vch.ca (F.D.); sgill@bccancer.bc.ca (S.G.);; 2 Department of Surgery, Division of General Surgery, St. Paul’s Hospital, Vancouver, BC V6Z1Y6, Canada; 3 Division of Gastroenterology, Vancouver General Hospital, Vancouver, BC V5Z1M9, Canada; 4 BC Cancer, Vancouver, BC V6E1Y6, Canada

**Keywords:** early onset colorectal cancer, colorectal cancer symptoms, screening

## Abstract

**Background**: The incidence of colorectal cancer (CRC) is decreasing in individuals >50 years due to organised screening but has increased for younger individuals. We characterized symptoms and their timing before diagnosis in young individuals. **Methods**: We identified all patients diagnosed with CRC between 1990–2017 in British Columbia, Canada. Individuals <50 years (*n* = 2544, EoCRC) and a matched cohort >50 (*n* = 2570, LoCRC) underwent chart review to identify CRC related symptoms at diagnosis and determine time from symptom onset to diagnosis. **Results:** Across all stages of CRC, EoCRC presented with significantly more symptoms than LoCRC (Stage 1 mean ± SD: 1.3 ± 0.9 vs. 0.7 ± 0.9, *p* = 0.0008; Stage 4: 3.3 ± 1.5 vs. 2.3 ± 1.7, *p* < 0.0001). Greater symptom burden at diagnosis was associated with worse survival in both EoCRC (*p* < 0.0001) and LoCRC (*p* < 0.0001). When controlling for cancer stage, both age (HR 0.87, 95% CI 0.8–1.0, *p* = 0.008) and increasing symptom number were independently associated with worse survival in multivariate models. **Conclusions**: Patients with EoCRC present with a greater number of symptoms of longer duration than LoCRC; however, time from patient reported symptom onset was not associated with worse outcomes.

## 1. Introduction

Colorectal cancer (CRC) is the third most common cancer in Canada [1]. While the incidence of CRC has decreased in older patients (≥50 years of age, LoCRC), there has been a rise in the incidence of CRC in patients <50 years of age (EoCRC) [2,3,4]. The decrease in incidence of CRC in older patients has been primarily attributed to organised screening removing pre-malignant polpys [1,5]. Current screening guidelines in Canada recommend screening for CRC begin at the age of 50 [6,7]. Recently, in response to the rising incidence of early-onset CRC, American organisations, such as the American Cancer Society and US Preventive Services Task Force, have updated their screening guidelines and recommend screening beginning at age 45 in average-risk adults [6]. 

As younger patients are excluded from screening programs and there is less awareness about the risk of CRC in young patients, disease in this population may not be detected until patients are more symptomatic and later in their disease course [8,9,10,11]. Possible explanations include patient-related factors, such as lack of knowledge about symptoms which may contribute to a longer time to diagnosis [12,13]. Provider-related factors have also been shown to play a role in delaying diagnosis where, despite younger patients presenting with symptoms that should prompt investigation of CRC, there may be a low suspicion among health care providers [14,15]. Given these issues, our objectives were to (1) characterize the pattern of symptoms at presentation in EoCRC compared to LoCRC, (2) identify differences in the number of symptoms at presentation and whether this associates with survival, and (3) assess if EoCRC patients experience a longer time to diagnosis compared to LoCRC and how this impacts survival.

## 2. Materials and Methods

### 2.1. Patient Population

Cancer care in the Canadian province of British Columbia (population ~5 million) is publicly funded and administered through 6 regional cancer centres under the guidance of BC Cancer. Within BC Cancer, the Gastrointestinal Cancer Outcomes Unit (GICOU) prospectively collects demographic, disease, treatment, and outcome data on all patients with CRC. After receiving institutional review board approval, the GICOU database was queried to identify patients diagnosed with CRC between 1990–2017. Patients diagnosed with CRC before age of 50 were deemed early-onset (EoCRC), while other patients were categorized as late-onset (LoCRC). There were 2544 in the EoCRC cohort and 27,616 in the LoCRC. 

All individuals in the EoCRC cohort, and a matched cohort of LoCRC patients who were diagnosed between 1 January 2015–31 December 2016 were included in a retrospective chart review (Table S1). The LoCRC subset was chosen from more recent dates of diagnosis to ensure maximum availability of hospital records to conduct our analysis and to ensure a minimum 5 years of follow up for comparison. We selected 18 symptoms associated with different stages of CRC, including early stage (anemia, change in bowel habits, decreased stool calibre, diarrhea, constipation, bloody stools); locally advanced (urinary symptoms, palpable mass, low back pain, tenesmus, nausea/vomiting, abdominal pain); and advanced/metastatic disease (night sweats, weight loss, fatigue, fever, ascites, anorexia) and ascertained if they were reported and how long before diagnosis they were reported. We included asymptomatic stool testing for occult blood and screening colonoscopy as “incidental” diagnoses.

### 2.2. Statistical Methods

Prevalence of symptoms and complications at presentation or during the index operation including obstruction, perforation and hemorrhage was compared with the Chi-square or Fisher’s exact test as appropriate and an odds ratio with 95% confidence interval was calculated. The number of symptoms at diagnosis grouped by stage was compared between EoCRC and LoCRC using Welch’s *t* test. Kaplan–Meyer curves were used to summarize overall survival and compared via the log-rank test. Hazard ratios for overall survival of each symptom were calculated by comparing survival of each listed symptom with bloody stools as the reference since it was the most common. The duration of symptoms was calculated from the time of symptom onset until the date of pathologic diagnosis based on chart review. The difference in median symptom duration prior to diagnosis was compared using the Mann–Whitney test. 

After all included variables were shown to satisfy the proportional hazards assumption, a Cox-proportional hazard model for overall survival was created using a forward likelihood-ratio inclusion method with *p* < 0.05 probability for stepwise inclusion and *p* > 0.10 probability for exclusion. Variables assessed included age, diagnosis era, stage at diagnosis, number of symptoms and duration of symptoms (categorical as 0–90 days, 91–180 days and 181+ days). A total of 3344 patients with all values available were analyzed.

## 3. Results

### 3.1. Baseline Characteristics

The baseline demographics of EoCRC and LoCRC are shown in Table 1. There is a slightly higher preponderance of males in the LoCRC cohort (EoCRC 51% vs. LoCRC 58%, *p* < 0.0001). The median age at diagnosis for the EoCRC cohort is 44 years, and 76 years for LoCRC.

The baseline demographics of the subgroup of patients reviewed for presence of symptoms compared to the whole group are shown in Table S1. There was a significant difference in the date of diagnosis and clinical stage. 

### 3.2. Symptom Distribution at Time of Cancer Diagnosis

**Figure 1:**
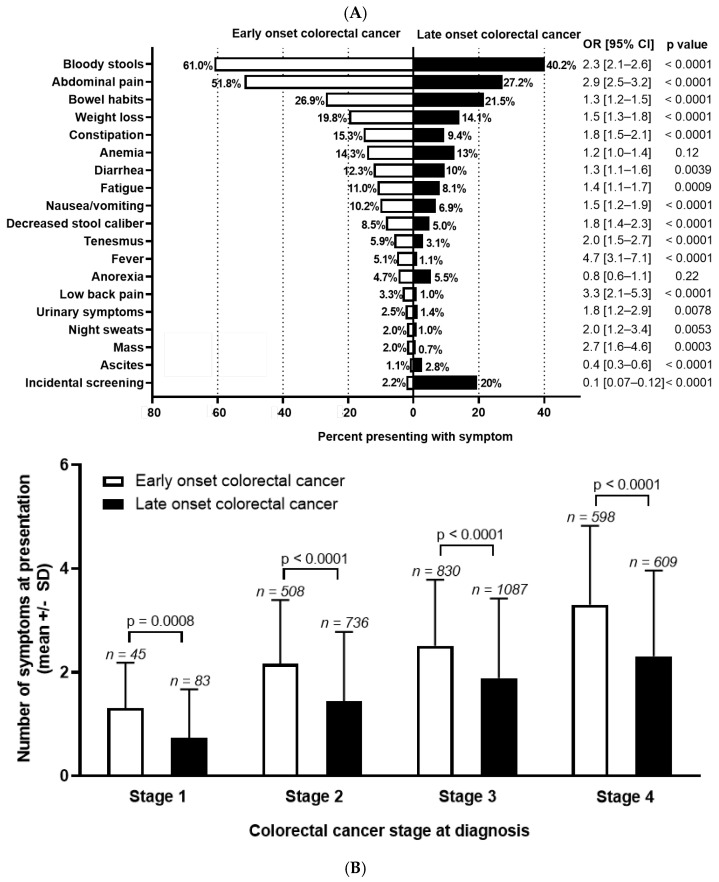
(**A**) Symptom distribution and (**B**) number at diagnosis for early-onset (patients under 50) and late-onset (patients 50 years or older) with colorectal cancer. Odds ratio (OR) and 95% confidence interval (CI) shown to the right of the graph and calculated using Fisher’s exact test. Stage 0 and undefined stage excluded from analysis. Symptom number at each stage of colorectal cancer calculated with unpaired *t* test.

Among patients who underwent chart reviews, sufficient history was present in 1992 EoCRC and 2504 LoCRC to assign symptoms preceding diagnosis. Differences in symptoms are presented in Figure 1A. The most common symptoms at presentation are bloody stools (EoCRC 61.0% vs. LoCRC 40.2%; OR 2.3 (95% CI 2.1–2.6), *p* < 0.0001); abdominal pain (EoCRC 51.8% vs. LoCRC 27.2%; OR 2.9 (95% CI 2.5–3.2), *p* < 0.0001); and change in bowel habits (EoCRC 26.9% vs. LoCRC 21.5%; OR 1.3 (95% CI 1.2–1.5)), *p* < 0.0001). When comparing symptoms between groups, EoCRC more commonly presented with nearly all symptoms. This is with the exception of anemia and anorexia, which were not significantly different, and ascites (*p* < 0.0001) which was more common in LoCRC. Diagnosis through screening for EoCRC was low, as expected, at 2.2%, mainly occurring in individuals with hereditary cancers or inflammatory bowel disease. For LoCRC, this was significantly higher at 19.6% (OR 0.09, 95% CI 0.07–0.12, *p* < 0.0001). 

Next, we compared the total number of symptoms by age, stratified for stage at diagnosis (Figure 1B). As stage increased, the number of symptoms at presentation increased in both groups (*p* < 0.0001 for both EoCRC and LoCRC). At all stages, EoCRC presented with significantly more symptoms than LoCRC (mean number of symptoms ± SD for Stage 1 EoCRC 1.3 ± 0.9 vs. LoCRC 0.7 ± 0.9, *p* < 0.001; Stage 2 EoCRC 2.2 ± 1.2 vs. LoCRC 1.4 ± 1.3, *p* < 0.001; Stage 3 EoCRC 2.5 ± 1.3 vs. LoCRC 1.9 ± 1.5, *p* < 0.001; Stage 4 EoCRC 3.3 ± 1.5 vs. LoCRC 2.3 ± 1.7, *p* < 0.001).

### 3.3. Association of Number of Symptoms at Diagnosis with Survival

**Figure 2:**
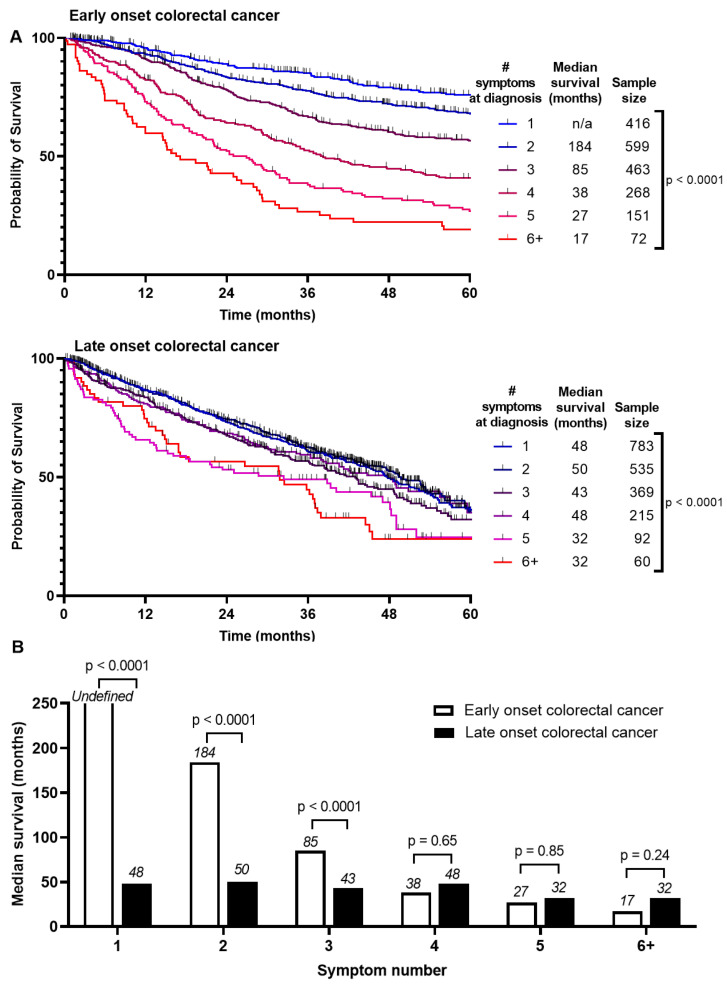
Comparison of survival by (**A**) number of symptoms at presentation of patients with early onset (patients under 50) and late onset (patients 50 years or older) with colorectal cancer and (**B**) summary of median survivals based on number of symptoms at presentation and log-rank test comparing entire survival curve.

Survival between EoCRC and LoCRC was divided into Stage 1–3 and Stage 4 CRC (Figure S2). Median survival is higher for EoCRC than LoCRC if presenting with Stage 1–3 (190 months vs. 53 months, *p* < 0.0001). There was no difference if presenting with Stage 4 (18 months vs. 17 months, *p* = 0.12). We asked whether these survival differences are associated with number of symptoms at presentation (Figure 2A). For EoCRC, median survival is shortened with increased number of symptoms at presentation from 184 months if presenting with 2 symptoms, 85 months if presenting with 3, and 17 months if presenting with 6 or more symptoms (*p* < 0.0001) (Figure 2B). Similar results were noted for LoCRC, to a lesser magnitude (*p* < 0.0001). There was little or no difference in disease free survival (Figure S3).

Next, we asked whether this difference in survival is due to higher symptom burden being associated with later stage at diagnosis and therefore a worse prognosis. We conducted separate subgroup analyses based on stage at diagnosis in early (stage 1–3) and metastatic (stage 4) CRC (Figure S4). Regardless of whether EoCRC present with early or metastatic disease, the pattern persists where greater symptom burden at presentation, is associated with worse survival (*p* < 0.0001). This same finding was also seen in LoCRC for both early (*p* = 0.001) and metastatic stage at diagnosis (*p* = 0.02). The magnitude of this difference is smaller than for the EoCRC cohort. 

### 3.4. Symptoms Associated with Advanced Stage Predict Worse Survival

**Figure 3:**
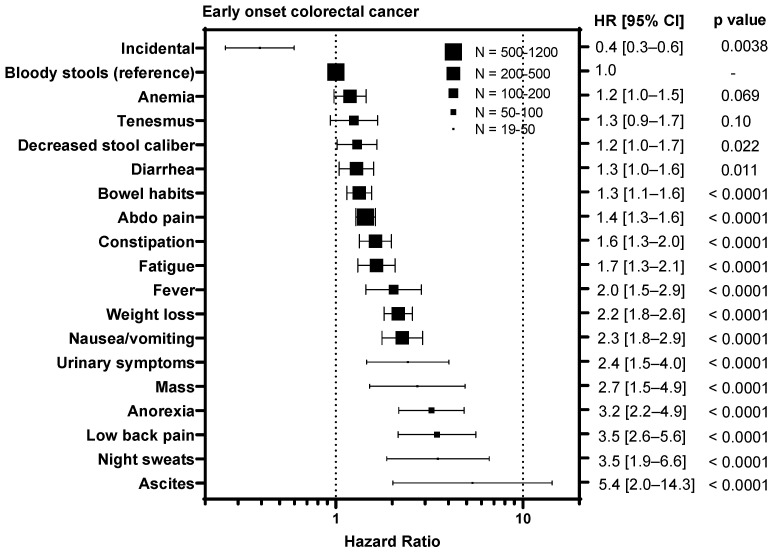

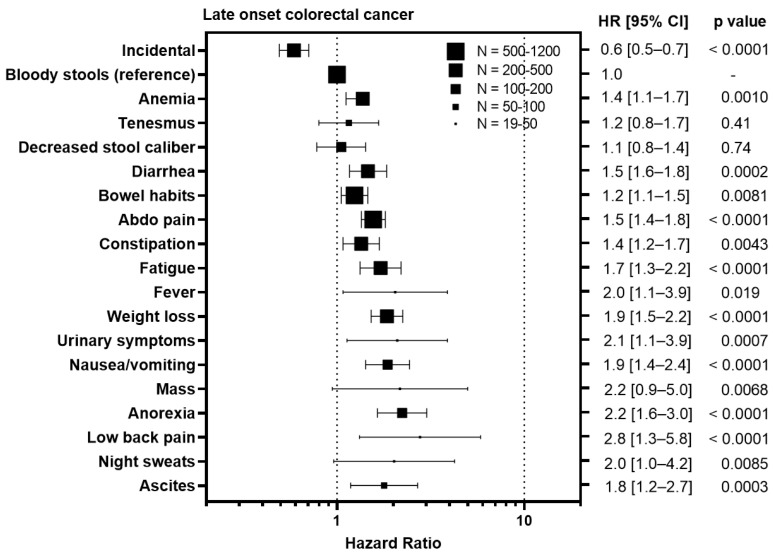
Impact of symptoms associated with colorectal cancer relative on overall survival to bloody stools in patients with early onset (patients under 50) and late onset (patients 50 years or older) with colorectal cancer. HR = hazard ratio.

To understand whether certain symptoms are associated with a worse prognosis, we calculated hazard ratios for each symptom compared to bloody stools, the most prevalent symptom (Figure 3). Diagnosis of CRC incidentally during screening colonoscopy/FIT test was associated with an improved outcome in EoCRC with a HR of 0.4 (95% CI 0.3–0.6, *p* = 0.0038) and LoCRC showing a HR 0.6 (95% CI 0.5–0.7, *p* < 0.0001). In contrast, symptoms associated with metastatic disease were associated with worse outcomes for both EoCRC and LoCRC. These included ascites (EoCRC HR 5.4, 95% CI 2.0–14.3, *p* < 0.0001; LoCRC 1.8, 95% CI 1.2–2.7, *p* = 0.0003); night sweats (EoCRC HR 3.5, 95% CI 1.9–6.6, *p* < 0.0001; LoCRC 2.0, 95% CI 1.0–4.2, *p* = 0.0085); fever (EoCRC HR 4.7, 95% CI 3.1–7.1, *p* < 0.0001; LoCRC 2.8, 95% CI 1.3–5.8, *p* < 0.0001); and anorexia (EoCRC HR 3.2, 95% CI 2.2–4.9, *p* < 0.0001; LoCRC 2.2, 95% CI 1.6–3.0, *p* < 0.0001). In contrast, symptoms associated with local tumor growth including tenesmus (EoCRC HR 1.3, 95% CI 0.9–1.7, *p* = 0.10; LoCRC 1.2, 95% CI 0.8–1.7, *p* = 0.41); decreased stool caliber (EoCRC HR 1.2, 95% CI 1.0–1.7, *p* = 0.022; 1.1, 95% CI 0.8–1.7, *p* = 0.74); and diarrhea (EoCRC HR 1.3, 95% CI 1.0–1.6, *p* = 0.011; LoCRC 1.5, 95% CI 1.6–1.8, *p* = 0.0002) are less or not associated with outcome.

### 3.5. Time from Symptom Onset to Diagnosis Is Longer for EoCRC

**Figure 4:**
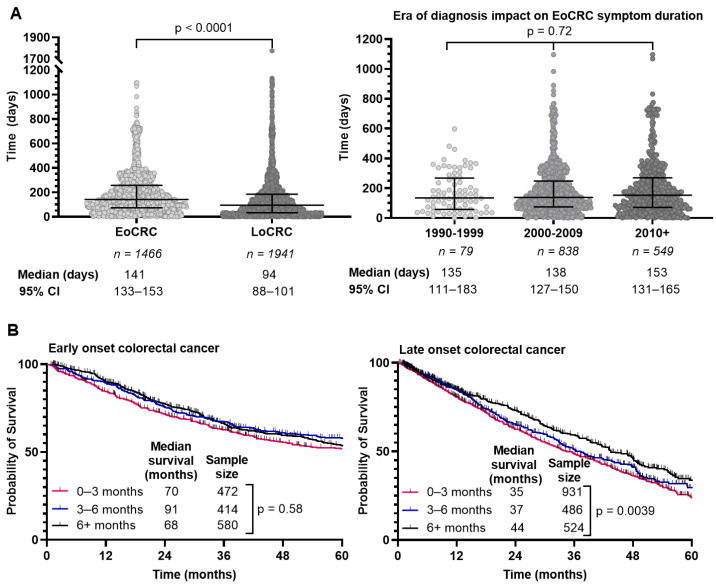
Duration of symptoms prior to colorectal cancer diagnosis (**A**), survival of patients with early-onset (patients under 50) and late-onset (patients 50 years or older) colorectal cancer by duration of symptoms (**B**). Duration of symptoms for each individual excluding inflammatory bowel disease compared using Kruskal–Wallis test (**A**).

We determined the interval from symptom onset to diagnosis through retrospective chart review where able and summarized the results in Figure 4A. EoCRC had a median interval of 143 days (95% CI 134–154) compared to 95 days (95% CI 88–101) in LoCRC (*p* < 0.0001). We hypothesized that delay in diagnosis of EoCRC may be mitigated in those diagnosed more recently given increased awareness of the rising incidence of CRC in younger individuals. We separated the population based on diagnosis year and evaluated the interval from symptom onset to diagnosis (Figure 4A, right). In EoCRC, a more recent diagnosis did not result in shorter duration of symptoms before diagnosis. If diagnosed after 2010, median time to diagnosis was 156 days (95% CI 137–169); between 2000–2009 the median time was 138 days (95% CI 126–151); and between 1990–1999 the median time was 134 days (95% CI 103–183) (*p* = 0.72).

Given the prolonged time to diagnosis in EoCRC compared to LoCRC, we asked whether that affected survival. We compared survival for EoCRC with symptom duration between 0–3, 3–6 and 6+ months (Figure 4B). For EoCRC, there was no difference in survival, with a median survival of 70 months for symptom duration 0–3 months, 91 months for 3–6 months, and 68 months for over 6 months (*p* = 0.58). In the LoCRC cohort, there was a difference in outcome based on symptom duration, with a median survival of 35 months for symptoms lasting 0–3 months, 37 months for 3–6 months, and 44 months for over 6 months (*p* = 0.0039).

**Figure 5:**
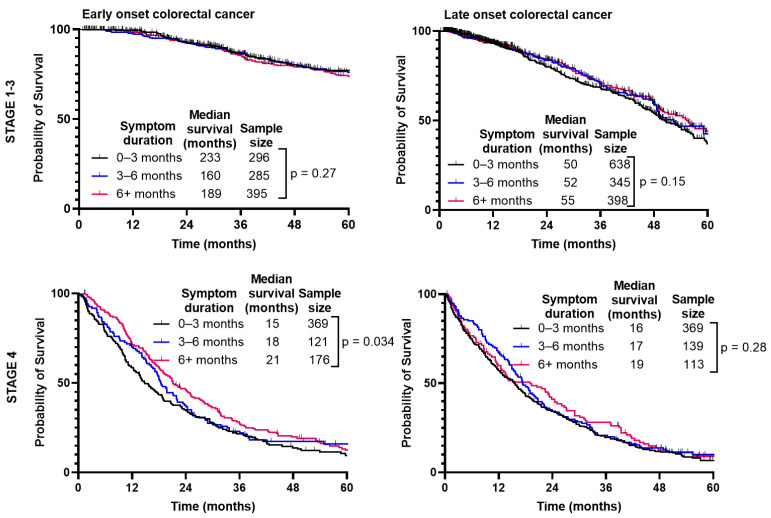
Survival of patients with early-onset (patients under 50) and late-onset (patients 50 years or older) colorectal cancer is not affected by duration from symptom onset to time of diagnosis.

When separated by CRC stage (Figure 5), there was no difference in median survival when comparing duration of symptoms for Stage 1–3 (*p* = 0.27); however, there was a slight improvement paradoxically with longer symptom duration for Stage 4 (*p* = 0.034). With LoCRC, there was no difference in survival for Stage 1–3 (*p* = 0.15) or Stage 4 (*p* = 0.28).

**Figure 6:**
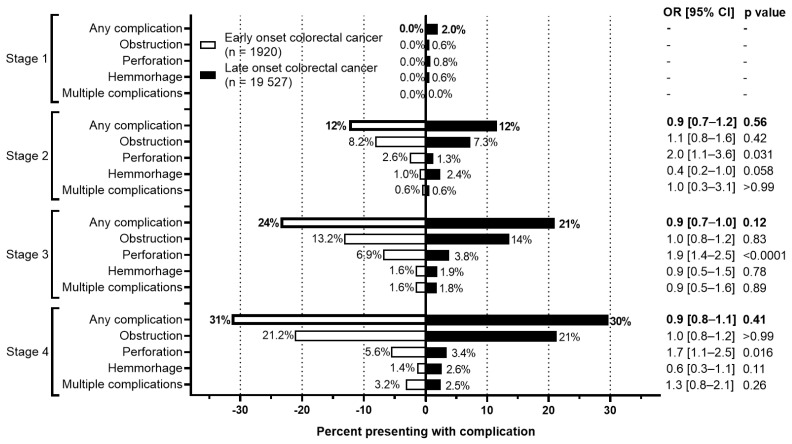
Rate of complications by stage of colorectal cancer. Odds ratio calculated using Fisher’s exact test.

The rate of complications at presentation or during the initial surgery between EoCRC and LoCRC were also evaluated (Figure 6). For both cohorts, more advanced stages were associated with higher rates of complications. For EoCRC, this increased from 0% at stage 1 to 31.4% at stage 4. Similarly, for LoCRC, this increased from 2.0% at stage 1 to 29.7% at stage 4. Between both EoCRC and LoCRC, however, there is no difference in the rate of complications at any stage of presentation.

### 3.6. Multivariate Analysis Shows Symptom Number, Age, and Stage of CRC Diagnosis Are Independent Predictors of Survival

To account for confounding variables within our known dataset we performed a multivariate analysis that assessed the impact of stage at diagnosis, age, duration of symptoms and symptom burden on outcome (Table S2). We noted that age < 50 years old (HR 0.87, 95% CI 0.78–0.96, *p* = 0.0080) and fewer number of symptoms at diagnosis are independent predictors of improved overall survival. However, duration of symptoms did not impact survival and did not meet criteria for inclusion in the model (see methods). 

## 4. Discussion

Our study reviewed patterns of presentation, diagnostic delays, and outcomes in EoCRC. Multiple studies have shown early-onset patients experience diagnostic delays and diagnosis at more advanced stages of CRC [8,16,17]. As individuals <50 fall outside screening programs in most countries, it is imperative to understand the constellation of symptoms they present with. Our study demonstrates that EoCRCs present at more advanced stages (although the difference is small in absolute numbers, it is significant due to the size of the database), have more symptoms at diagnosis, and experience diagnostic delays, however in our dataset these delays did not result in worse survival. Furthermore, the number of symptoms at presentation plays a role in determining the survival of all individuals with CRC, but to a much greater extent in EoCRC. This persists after controlling for confounding variables with multivariate analysis.

The pattern of symptoms at presentation is similar between EoCRC and LoCRC. The three most common symptoms are bloody stools, abdominal pain and change in bowel habits, consistent with prior studies [18,19,20]. Several prospective studies [21,22] and a systematic review [23] to determine the positive predictive value of CRC-type symptoms found that these three symptoms individually and in combination are effective at predicting CRC. While it is important to recognize that these three symptoms may be indicative of other benign diseases, placing CRC on the differential is imperative, especially in younger individuals.

Our group found EoCRC has improved survival compared to LoCRC, similar to the work of others [10,24,25,26]. Despite this, the interval duration from symptom onset to diagnosis was longer in EoCRC, in accordance with multiple studies [27,28]. Multiple reasons for this have been postulated, including lack of recognition of symptom significance and denial on both the part of the clinician and patient [4,29]. We have also shown in this study, that duration of symptoms does not impact survival significantly, which is a key finding. In this case, what is driving this survival advantage in EoCRC? We found a strong predictor of survival is the symptom burden at diagnosis and noted that young patients presented with more symptoms. This may be a reflection of a more aggressive tumor causing rapid growth for patients with symptoms, while other young patients may have slower growing tumors and their pre-morbid health may be protective and improve the median survival of the overall cohort. 

Our study has important limitations. First, symptoms were collected through retrospective chart review of a pre-defined list of symptoms, some of which may not have been documented, and therefore our analysis may under-represent symptom burden. In addition, our selection of the LoCRC cohort from more recent years (2015–2016) could have introduced a survival bias relative to the EoCRC cohort (1990–2017), as there have been advancements in awareness, screening adherence, and treatment. Second, the duration of symptoms is subject to recall bias as this information was collected from documents at the time of diagnosis, limiting the interpretation of the absolute median days from symptom onset to diagnosis. Third, we found that LoCRC had a rate of 20% detection of cancer through screening. The uptake in BC’s Colon Screening Program in 2019 shows a participation rate of 30–39%, depending on age and a recent randomized study suggested uptake in a clinical trial was only 42% [1]. Overall, uptake in screening programs is low, and with this study we demonstrate the benefit to survival of identifying CRC prior to development of symptoms. 

In conclusion, we demonstrate the importance of recognizing symptoms associated with CRC. While symptom burden was associated with survival, we did not note delays in CRC diagnosis as a predictor of survival or complications during initial presentation. With the rising incidence of EoCRC, more work is needed to understand how best to identify these patients early, so they have the best chance of cure.

## Figures and Tables

**Table 1:** Baseline characteristics of individuals diagnosed with colorectal cancer between 1990–2017 in British Columbia, Canada compared between those less than 50 (EoCRC) or 50 years and older (LoCRC) with colorectal cancer. The absolute number of individuals (*n*), proportion of the specified cohort (%) and *p* values are shown.

Characteristics	Early-Onset Colorectal Cancer		Late-Onset Colorectal Cancer		*p* Value
	*n*	%	*n*	%	
**Sex**					
Male	1308	51.4	15,979	57.9	<0.0001
Female	1236	48.6	11,637	42.1	
**Median age at diagnosis (min–max)**	44 (14–49)		76 (50–104)		<0.0001
**Diagnosis date**					
1990–1994	288	11.3	2126	8.5	<0.0001
1995–1999	340	13.4	2906	11.6	
2000–2004	488	19.2	4629	18.5	
2005–2009	529	20.8	5881	23.5	
2010–2014	655	25.7	6906	27.5	
2015–2017	244	9.6	2624	10.5	
**Clinical stage**					
0	4	0.2	62	0.2	<0.0001
1	55	2.2	610	2.4	
2	668	26.3	7609	30.3	
3	1015	39.9	9897	39.5	
4	734	28.9	6030	24.1	
Unknown	68	2.7	864	3.4	
**Histology**					
Adenocarcinoma	2514	98.8	24,679	98.4	0.0006
Mucinous cell adenocarcinoma	152	6.0	1478	6.0	
Signet ring cell adenocarcinoma	32	1.3	150	0.6	
Other	30	1.2	393	1.6	

## Data Availability

The datasets generated and/or analysed during the current study are not publicly available due to patient privacy but are available from the corresponding author on reasonable request.

## Supplementary Materials

The following supporting information can be downloaded at: https://www.mdpi.com/article/10.3390/curroncol31040158/s1, Figure S1: Consort diagram of subset cohort of patients younger than 50 (EoCRC) and 50 years or older (LoCRC) with colorectal cancer. Table S1. Demographic characteristics of all individuals ≥ 50 yo and a matched subset of the ≥50 yo cohort (LoCRC). Figure S2. Median survival of patients younger than 50 (EoCRC) and 50 years or older (LoCRC) by stage of cancer at presentation. Figure S3. Disease free survival of patients with early-onset (patients under 50) and late-onset (patients 50 years or older) colorectal cancer by number of symptoms at presentation. Figure S4. Survival of patients with early-onset (patients under 50) and late-onset (patients 50 years or older) with colorectal cancer by number of symptoms at presentation, separated by stage of CRC at presentation. Figure S5. Median survival of patients younger than 50 (EoCRC) and 50 years or older (LoCRC) by symptom number at presentation and stage of diagnosis of colorectal cancer. Table S2. Multivariate analysis of the listed variables controlling for age, diagnosis era, stage at diagnosis, number of symptoms and duration of symptoms (categorical as 0–90, 91–180 and 181+ days).
