# Supplementary material for: Symptom Burden and Time from Symptom Onset to Cancer Diagnosis in Patients with Early-Onset Colorectal Cancer: A Multicenter Retrospective Analysis
Source: Curr Oncol. 2024 Apr 8;31(4):2133–44. doi: 10.3390/curroncol31040158 (PMC11049268; doi:10.3390/curroncol31040158)
Supplement: Supplementary file 1 [file curroncol-31-00158-s001.zip › curroncol-2892256-supplementary.pdf]

Figure S1. Consort diagram of subset cohort of patients younger than 50 (EoCRC) and 50 years or older (LoCRC) with colorectal cancer.

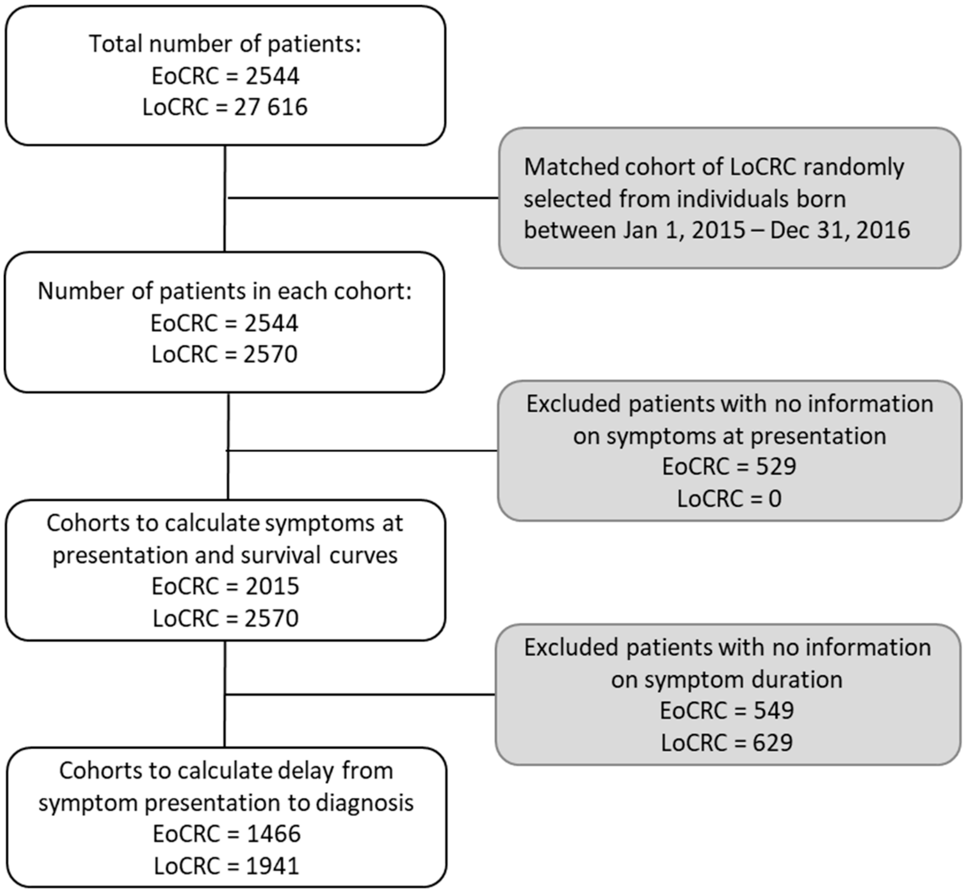

Table S1. Demographic characteristics of all individuals ≥ 50 yo and a matched subset of the ≥50 yo cohort (LoCRC).

Characteristics	LoCRC (subset)		LoCRC (all)		p value
	n	%	n	%	
**Sex**					
Male	1474	57.4	15979	57.9	0.63
Female	1096	42.6	11637	42.1	
**Median age at diagnosis (min-max)**	69 (50-104)		66 (50-104)		0.23
**Diagnosis date**					
1990-1994	0	0.0	2126	8.5	<0.0001
1995-1999	0	0.0	2906	11.6	
2000-2004	0	0.0	4629	18.5	
2005-2009	0	0.0	5881	23.5	
2010-2014	1	0.0	6906	27.5	
2015-2017	2569	100.0	2624	10.5	
**Clinical stage**					
0	5	0.2	62	0.2	<0.0001
1	83	3.2	610	2.4	
2	736	28.6	7609	30.3	
3	1087	42.3	9897	39.5	
4	609	23.7	6030	24.1	
Unknown	50	1.9	864	3.4	
**Histology**					
Adenocarcinoma	2523	98.2	24679	98.4	0.33
Mucinous cell	120	4.7	1478	6.0	
Signet cell	21	0.8	150	0.6	
Other	47	1.8	393	1.6	

Figure S2. Median survival of patients younger than 50 (EoCRC) and 50 years or older (LoCRC) by stage of cancer at presentation. Kaplan Meyer curves of all EoCRC and LoCRC divided by Stage 1-3 and Stage 4 excluding stage 0 and undefined stage**.**

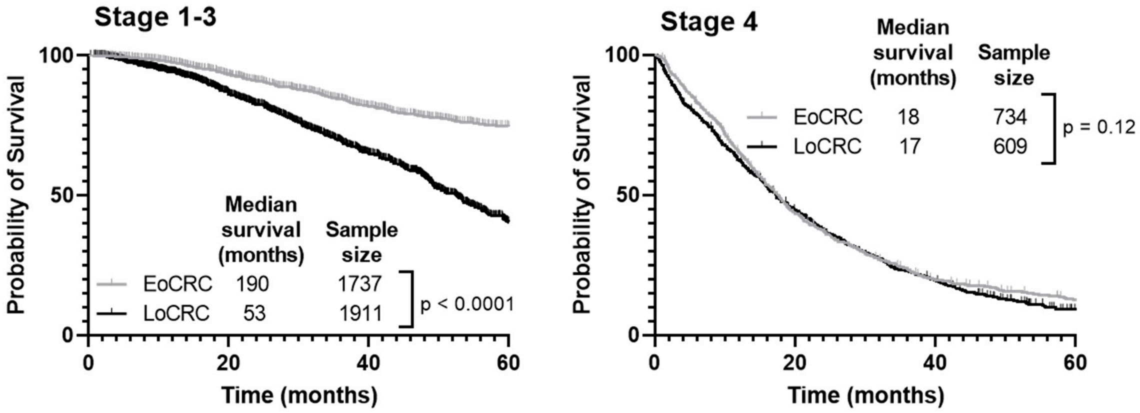

Figure S3. Disease free survival of patients with early-onset (patients under 50) and late-onset (patients 50 years or older) colorectal cancer by number of symptoms at presentation. Kaplan Meyer curves for EoCRC and LoCRC, all median survival values are undefined. Stage 0 and undefined stage excluded.

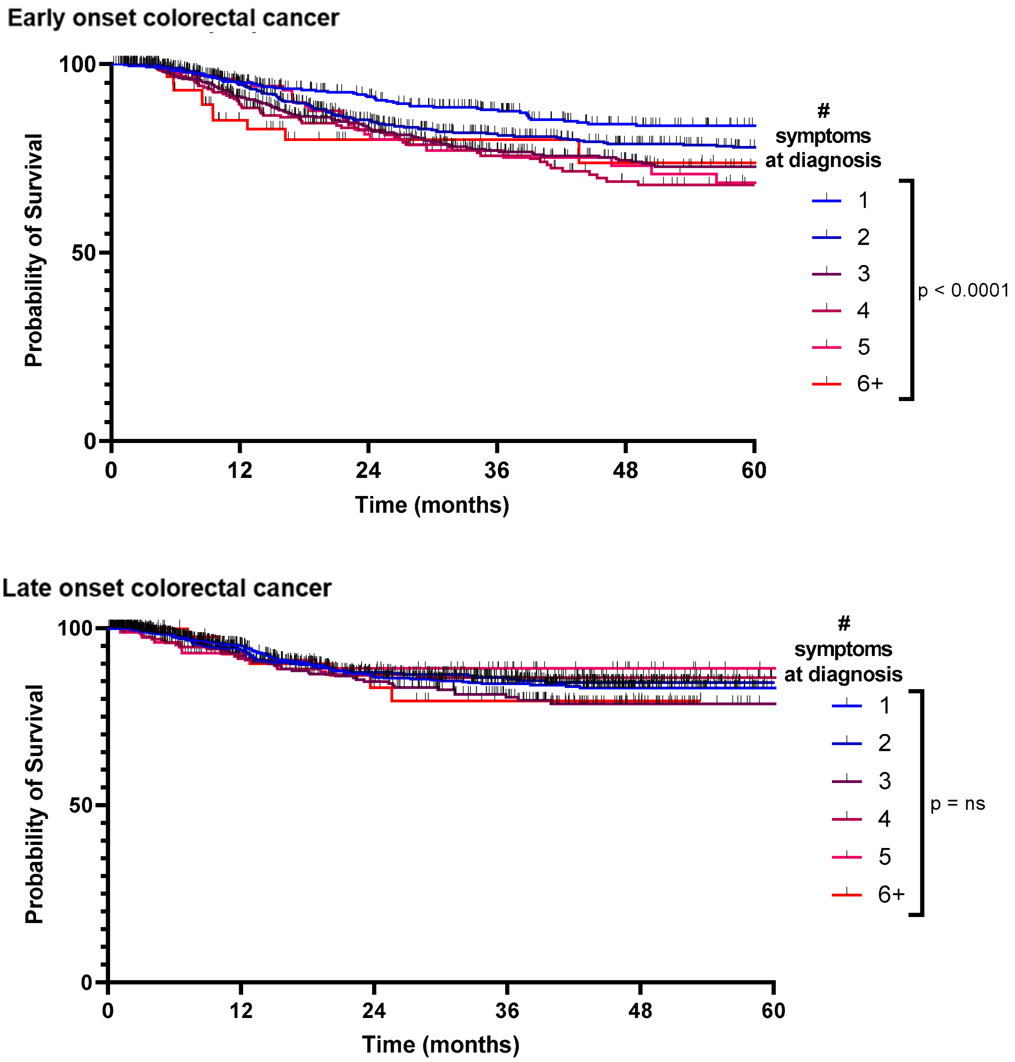

Figure S4. Survival of patients with early-onset (patients under 50) and late-onset (patients 50 years or older) with colorectal cancer by number of symptoms at presentation, separated by stage of CRC at presentation. Kaplan Meyer curves for EoCRC and LoCRC from Figure 3 are divided by either stage 1-3 or stage 4 CRC. Stage 0 and undefined stage excluded.

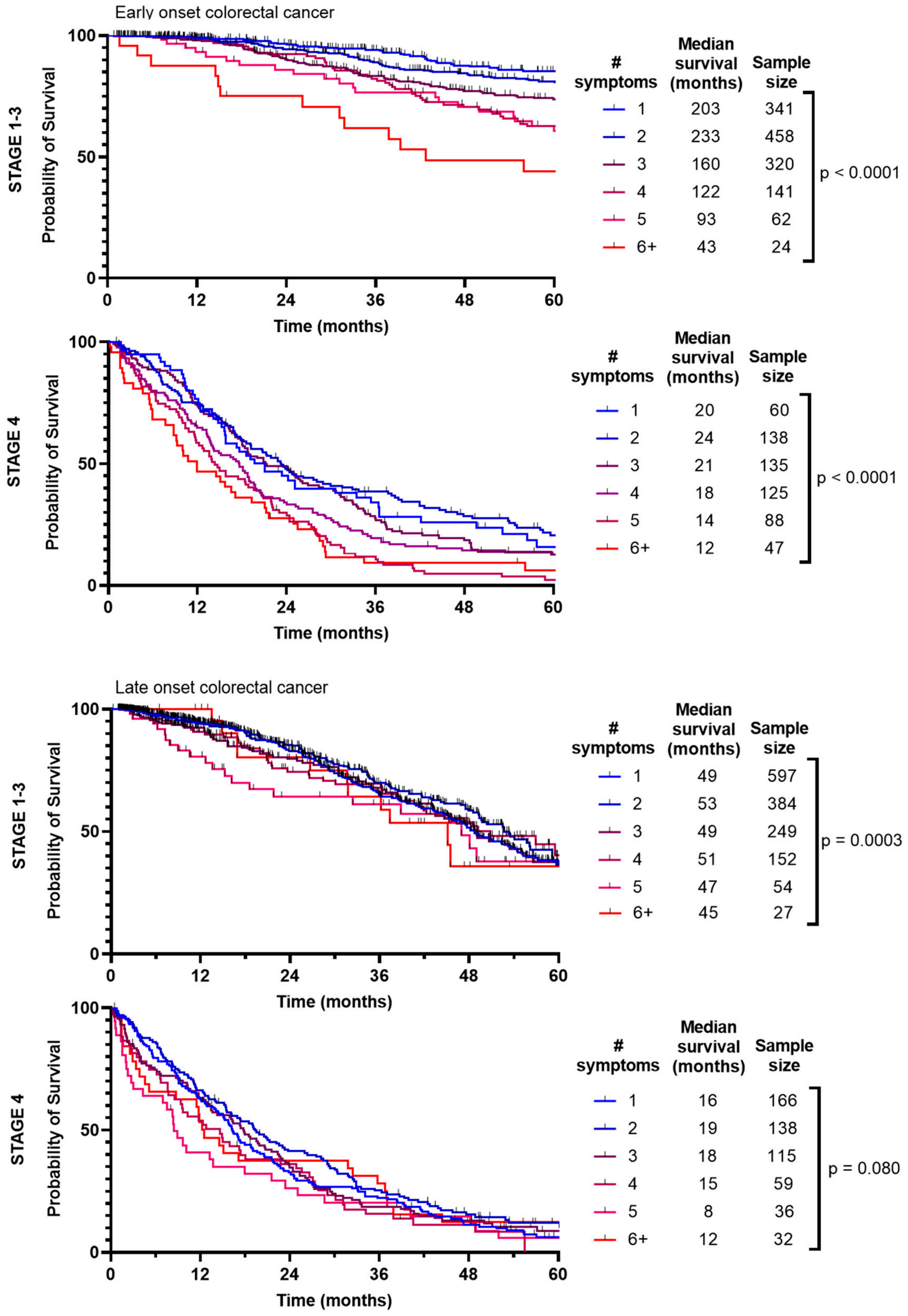

Figure S5. Median survival of patients younger than 50 (EoCRC) and 50 years or older (LoCRC) by symptom number at presentation and stage of diagnosis of colorectal cancer. Median survival calculated from Kaplan Meyer curves in Supplementary Figure 2, and p-value summarizes log-rank comparisons.

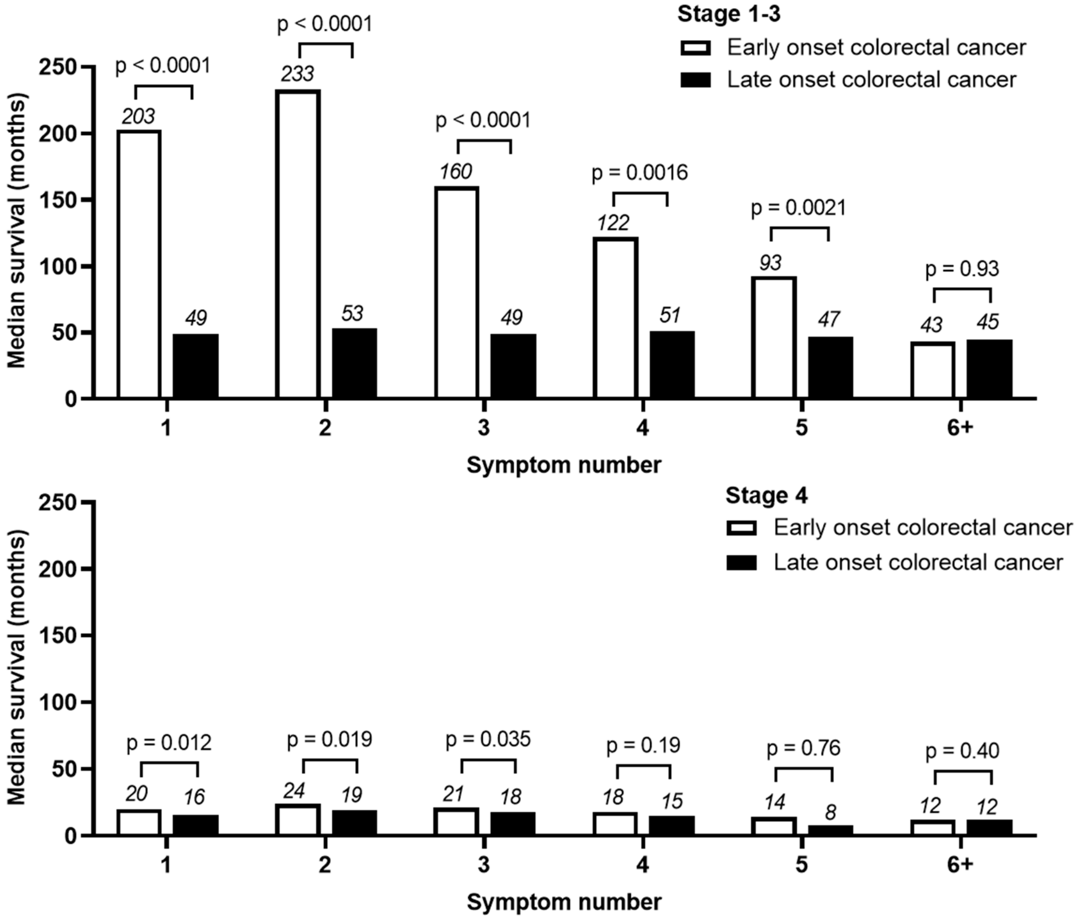

Table S2. Multivariate analysis of the listed variables controlling for age, diagnosis era, stage at diagnosis, number of symptoms and duration of symptoms (categorical as 0-90, 91-180 and 181+ days). A total of 3344 patients with all available values were analyzed using a Cox-proportional hazard model.

Variable	HR (95% CI)	P
Age<50	0.87 (0.78-0.96)	0.008
**Stage**		
Stage 1	0.12 (0.066-0.23)	<0.0001
Stage 2	0.11 (0.090-0.13)	<0.001
Stage 3	0.25 (0.23-0.28)	<0.001
Stage 4	Reference	
**Total Number of Symptoms**		
0	Reference	
1	1.48 (1.12-1.95)	0.006
2	1.36 (1.027-1.80)	0.032
3	1.68 (1.27-2.23)	<0.0001
4	1.93 (1.44-2.58)	<0.0001
5	2.51 (1.84-3.43)	<0.0001
6	2.36 (1.63-3.42)	<0.0001
7	2.31 (1.46-3.68)	<0.0001
8	9.02 (3.27-24.88)	<0.0001
9	5.20 (0.72-37.58)	0.1
